# Intraprocedural re-rupture during endovascular treatment of ruptured intracranial aneurysms: A single-center experience

**DOI:** 10.1007/s00234-026-04022-8

**Published:** 2026-05-20

**Authors:** Mustafa Ismail, Norito Kinjo, Ariana Chacon, Sami Al Kasab, Alejandro M. Spiotta

**Affiliations:** Division of Neuroendovascular Surgery, Department of Neurosurgery, Medical University of South Carolina, Charleston, SC USA

**Keywords:** Intraprocedural re-rupture, Ruptured intracranial aneurysm, Coil embolization, Hunt–Hess grade, Vasospasm

## Abstract

**Background:**

Intraprocedural re-rupture (IPR-RR) of ruptured intracranial aneurysms is rare but often catastrophic. We aimed to quantify the incidence of IPR-RR at our institution and describe its clinical characteristics, procedural context, and outcomes.

**Methods:**

Endovascular treatments for ruptured aneurysms at a tertiary center were reviewed. Intraprocedural re-rupture (IPR-RR) was defined as angiographic contrast extravasation or extrusion of a device tip beyond the aneurysm wall. The primary objective was to characterize the incidence of IPR-RR and identify associated clinical and procedural factors. Secondary endpoints included in-hospital mortality and discharge functional status as measured by the modified Rankin Scale (mRS 0–2 vs. > 2).

**Results:**

IPR-RR occurred in 17/702 cases (2.4%). Patients with IPR-RR were more frequently aged 18–40 years (35.3% vs. 12.2%, *p* = 0.011) and had aneurysms > 10 mm (18% vs. 4%). Vascular risk factors, including hypertension, diabetes, hyperlipidemia, and smoking history, were similarly distributed between groups. Balloon-assisted coiling was used in 82.4% vs. 58.0% (*p* = 0.114). Favorable discharge outcome occurred in 29.4% vs. 51.5% (*p* = 0.072), while in-hospital mortality was 17.6% vs. 10.2% (*p* = 0.580).

**Conclusions:**

Intraprocedural re-rupture occurred in 2.4% of ruptured aneurysm treatments and was associated with worse early outcomes. Effective management relied on immediate coil packing, balloon inflation, and anticoagulation reversal. Procedural preparedness, including strict blood pressure control and readiness for cerebrospinal fluid diversion, is essential to limit secondary neurological injury in this rare but serious complication.

**Supplementary information:**

The online version contains supplementary material available at https://doi.org/10.1007/s00234-026-04022-8.

## Highlights

**What is already known:**

Intra-procedural re-rupture (IPR-RR) during endovascular treatment of ruptured intracranial aneurysms is rare but often devastating, with reported rates ranging from 7–15%. Prior studies have identified small aneurysm size, anterior communicating artery location, high Hunt–Hess grade, and balloon-assisted coiling as potential risk factors, but findings remain inconsistent.

**What this study adds:**

In a 12-year single-center cohort (n=702), IPR-RR occurred in 2.4% of cases. The event was associated with lower rates of favorable discharge outcome and higher need for CSF diversion across Hunt–Hess strata. Immediate coil packing and anticoagulation reversal were performed in all cases, with balloon inflation used in 82%.

**Impact on the field:**

These findings emphasize the importance of procedural preparedness, rapid team coordination, and structured rescue protocols to limit secondary neurological injury during this uncommon but serious complication.

**Supplementary information:**

The online version contains supplementary material available at https://doi.org/10.1007/s00234-026-04022-8.

## Introduction

Intraprocedural rupture (IPR) is a feared complication during endovascular treatment of intracranial aneurysms, especially with previously ruptured aneurysms. Unlike IPR during coiling of unruptured aneurysms, which has a lower incidence and better outcomes, intraprocedural re-rupture (IPR-RR) in ruptured cases is associated with higher morbidity and mortality due to a compromised cerebrovascular environment and reduced physiological reserve [1, 2]. The incidence of IPR during coiling is reported at 2–5% for unruptured aneurysms [3] but rises to 7–15% for ruptured ones, strongly linked to poor outcomes like symptomatic vasospasm, hydrocephalus requiring CSF diversion, and longer hospitalization [1, 4].

Recent research has focused on identifying predictors of IPR-RR during the coiling of ruptured aneurysms to guide procedural strategies and reduce risk. Significant independent risk factors include smaller aneurysm size, location at the anterior communicating artery, a high Hunt–Hess grade, and the use of balloon-assisted coiling [4–6]. Additionally, very small aneurysms—specifically those less than 4 mm in diameter—exhibit a fivefold increase in IPR risk during coiling, particularly within the anterior communicating artery (AComA) segment [7]. More recent evidence, however, indicates that contemporary techniques have improved the safety of very small AComA aneurysms, with overall IPR rates approaching those of larger aneurysms [8]. Despite these advances, contemporary data on the real-world incidence of IPR-RR and its peri-procedural impact in modern practice remain limited, particularly regarding how this complication influences early outcomes and is managed in real time. Given the clinical significance of this event, we sought to determine the incidence of IPR-RR at our institution and characterize its clinical presentation, procedural context, and early outcomes.

## Methods

### Study design and patient selection

We maintain a registry for aneurysms prospectively, and it is approved by the Institutional Review Board of the Medical University of South Carolina (MUSC) (IRB ID: Pro00090704). This study was conducted according to that, and informed consent was waived due to its retrospective design. The neuro-interventional database, maintained prospectively, was reviewed for all endovascular procedures carried out for ruptured intracranial aneurysms from January 1, 2013, to December 31, 2024. Inclusion criteria comprised patients aged 18 or older who presented with ruptured intracranial saccular aneurysms and received any endovascular treatment. Exclusion criteria included re-treatments of previously coiled or clipped lesions and cases with incomplete data. Out of 2565 procedures, 702 were included as index procedures. The primary endpoint (IPR-RR) was defined as either angiographic contrast extravasation or the visualization of a device tip outside the aneurysm wall. Device-related perforation was only included when accompanied by angiographic or procedural evidence consistent with rupture. The final analytic cohort consisted of 17 IPR-RR cases and 684 controls. This study was conducted and reported following the STROBE (Strengthening the Reporting of Observational Studies in Epidemiology) guidelines for cohort studies [9]. Aneurysm size was categorized using standardized definitions by Merritt et al. [10], with aneurysms larger than 10 mm classified as large.

### Data collection and variable definitions

For each procedure, patient demographics were extracted—age (18–40, 40–60, or 60–90 years) and sex—alongside vascular risk factors including hypertension, diabetes, hyperlipidemia, smoking status (never, prior, or current), and family history of intracranial aneurysm. Clinical severity at presentation was documented with the Hunt–Hess (HH) grade. Aneurysm details included maximum diameter (as a continuous measurement and size category), anatomical location (anterior communicating artery, posterior communicating artery, middle cerebral bifurcation, basilar apex, or other), morphology (saccular vs. fusiform/blister), and presumed etiology (idiopathic, dissecting, infectious, traumatic, or other). Treatment strategy included balloon-assisted coiling, stent-assisted coiling, flow diversion, simple coiling, or alternative techniques. The primary outcome was the incidence of IPR-RR. Secondary outcomes included in-hospital mortality and discharge functional status assessed using the modified Rankin Scale (mRS), dichotomized as favorable (0–2) and unfavorable (> 2).

### Statistical analysis

Continuous variables are reported as mean ± standard deviation (SD) or median [inter-quartile range] if they are non-normally distributed, while categorical variables are expressed as counts and percentages. Pearson χ² or Fisher’s exact tests were utilized to compare categorical variables, and Welch’s t-test was employed for continuous variables that met the normality assumption; otherwise, the Mann–Whitney U test was applied. Two-sided *p* < 0.05 was considered significant. All analyses were conducted in IBM SPSS Statistics, version 30.

## Results

### Baseline profile and frequency of IPR-RR

**Fig. 1:**
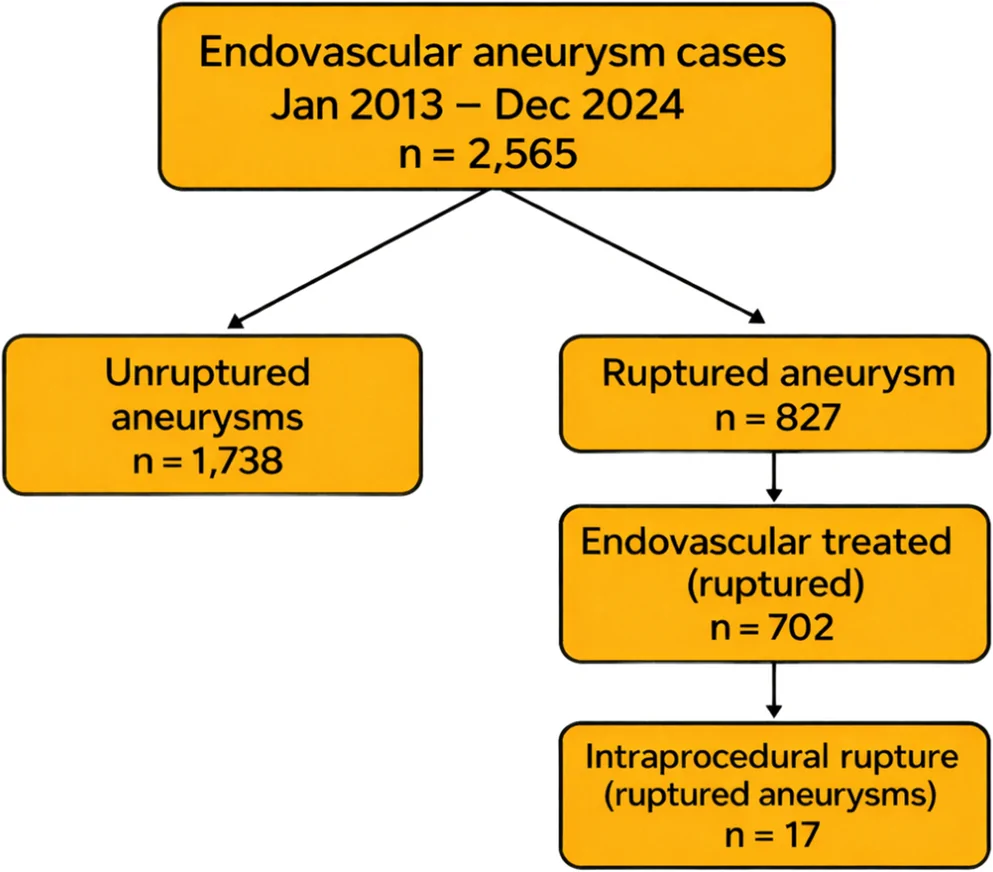
Flow diagram of endovascular aneurysm cases showing cohort stratification into unruptured and ruptured aneurysms, with further selection of endovascularly treated ruptured cases and occurrence of IPR-RR

**Fig. 2:**
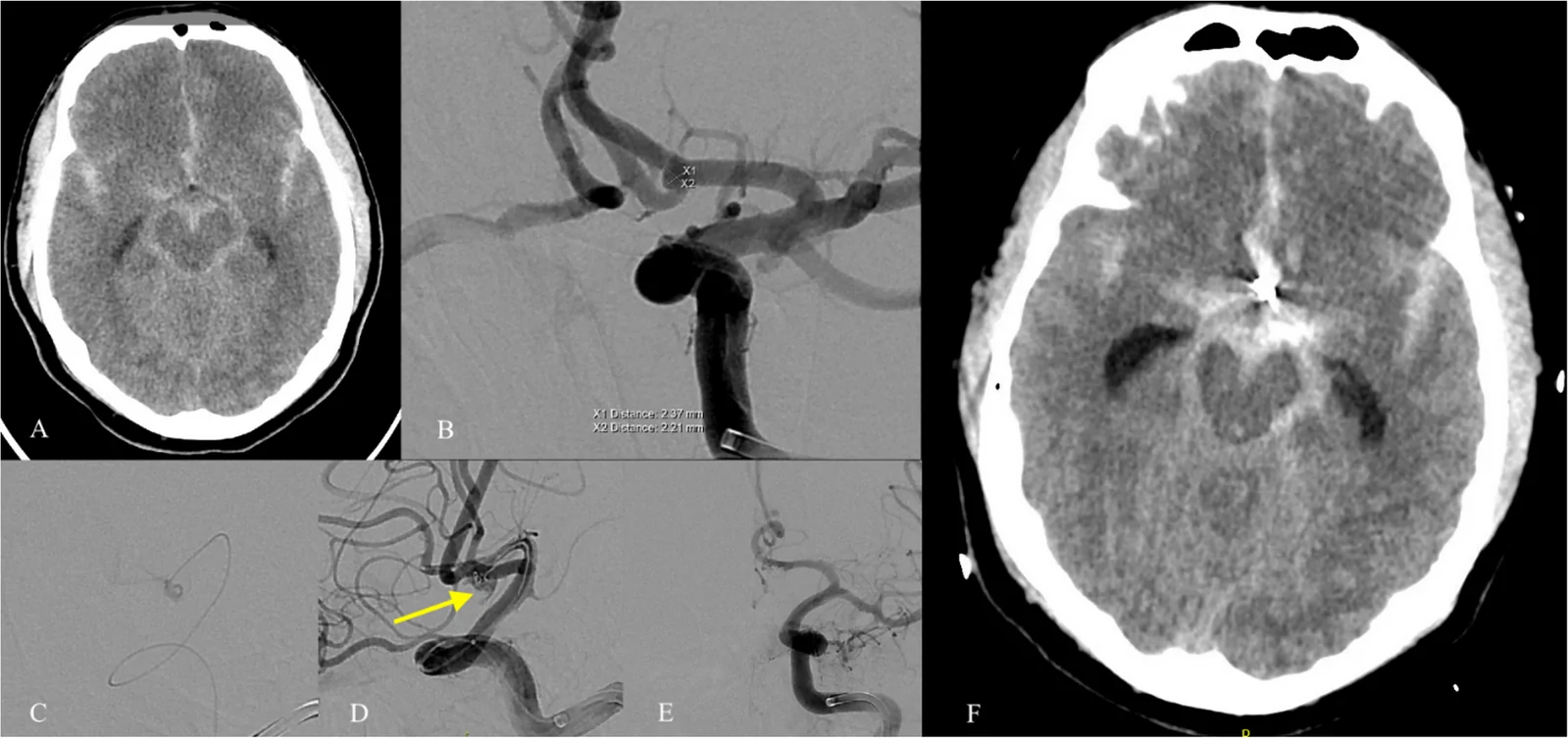
A 25-year-old male smoker presented with acute subarachnoid hemorrhage (SAH). (**A**) Initial non-contrast head CT demonstrated diffuse acute SAH. (**B**) Diagnostic cerebral angiography identified a small saccular anterior communicating artery (ACOM) aneurysm, measuring approximately 2.4 × 2.2 mm, with a smooth contour arising from the median ACOM complex. (**C**) Early intraprocedural fluoroscopy during endovascular access demonstrated stable catheter positioning prior to coil deployment. (**D**) During coil embolization, angiography demonstrated contrast extravasation consistent with intraprocedural aneurysm re-rupture (Yellow arrow). A balloon was already positioned across the aneurysm neck, allowing for immediate flow arrest and hemostatic control. (**E**) Subsequent angiographic runs following balloon inflation and continued coil packing demonstrated cessation of contrast extravasation with preservation of the parent vessels. (**F**) Post-procedural non-contrast CT confirmed interval redistribution and progression of subarachnoid hemorrhage, consistent with the intraprocedural re-rupture event

The cohort had a mean age of 56.2 ± 13.6 years, comprising 494 females (70.4%) and 207 males (29.5%). Seventeen of 702 coil-treated aneurysms experienced an IPR-RR (2.4%) **(**Fig. 1**)**. Patients who re-ruptured were significantly younger (35% vs. 12% aged 18–40 years; *p* = 0.011) and more frequently had large (> 10 mm) aneurysms (18% vs. 4%). Classic vascular risk factors—including hypertension, diabetes, hyperlipidemia, and smoking—were similarly distributed between the groups, while balloon-assisted coiling (BAC) was utilized in 82% of IPR-RR cases compared to 58% of uncomplicated cases. Functional outcomes diverged early: only 29% of IPR-RR patients were discharged with mRS ≤ 2, versus 52% of controls, although in-hospital mortality was not statistically different (18% vs. 10%, *P* = 0.580) **(**Table 1**)**. Figure 2 displays a case example of IPR-RR.

**Table 1:** Baseline, aneurysm, treatment and early-outcome characteristics stratified by intraprocedural re-rupture (IPR-RR)

Domain Variable	Category	IPR-RR (*n *= 17)	No IPR-RR (*n* = 685)	*p*-value†
Demographics				
Age group (yrs)	18 – 40	6 (35.3 %)	83 (12.2 %)	0.01
	40 – 60	8 (47.1 %)	323 (47.2 %)	
	60 – 90	3 (17.6 %)	275 (40.4 %)	
Sex	Female	13 (76.5 %)	481 (70.2 %)	0.58
	Male	4 (23.5 %)	203 (29.6 %)	
Vascular risk factors				
Hypertension	Yes	9 (52.9 %)	393 (57.4 %)	0.8
Diabetes mellitus	Yes	2 (11.8%)	88 (12.9 %)	0.9
Hyperlipidemia	Yes	2 (11.8%)	154 (22.5 %)	0.3
Smoking history	(current + former)	10 (58.8 %)	377 (55.1 %)	0.7
Family history of aneurysm	Yes	2 (11.8%)	44 (6.4 %)	0.3
Aneurysm location‡	ACOM	6 (35.3 %)	180 (26.3 %)	0.6
(top 4 sites)	PCOM	5 (29.4 %)	138 (20.1 %)	
	MCA bifurcation	2 (11.8%)	65 (9.5 %)	
	Basilar apex	0 (0 %)	49 (7.2 %)	
Aneurysm shape	Saccular	16 (94.1 %)	636 (92.8 %)	0.9
	Fusiform	1 (5.9 %)	37 (5.4 %)	
	Blister	0 (0 %)	11 (1.6 %)	
	Other/unknown	0 (0 %)	1 (0.1 %)	
Etiology	Idiopathic	16 (94.1 %)	613 (89.5 %)	0.9
	Dissecting / pseudo	1 (5.9 %)	54 (7.9 %)	
	Infectious	0 (0 %)	12 (1.8 %)	
	Traumatic	0 (0 %)	5 (0.7 %)	
	Other	0 (0 %)	1 (0.1 %)	
Treatment strategy	Balloon-assisted coiling (BAC)	14 (82.4 %)	397 (58.0 %)	0.1
	Stent-assisted coiling (SAC)	0 (0 %)	50 (7.3 %)	
	Other techniques§	3 (17.6 %)	238 (34.7 %)	
Early outcomes	In-hospital mortality	3 (17.6 %)	70 (10.2 %)	0.6
Functional status at discharge	Favorable (mRS ≤ 2)	5 (29.4 %)	353 (51.5 %)	0.07
	Unfavorable (mRS > 2)	12 (70.6 %)	332 (48.5 %)	

† Pearson χ² test comparing full category distributions (exact where appropriate)

‡ Remaining 21 anatomical sites pooled as “Other” (36.8 % No IPR-RR; 23.5 % IPR-RR)

§ Includes simple coiling, flow diversion, web devices, or combined/adjunctive approaches

### Peri-procedural course by HH grade and IPR-RR status

Across all HH strata, re-rupture worsened the peri-procedural course. In low-grade SAH (HH 1–2) without IPR-RR, two-thirds of patients achieved a favorable discharge mRS, and external ventricular drainage (EVD) was needed in only one-third. With IPR, the favorable outcome dropped to 43% (3/7), and EVD use increased to 43%. Similar trends were observed for vasospasm: radiographic vasospasm affected 25–27% of low-grade, no-IPR-RR cases but 50% of low-grade IPR cases. High-grade SAH (HH 4–5) without IPR-RR had high morbidity (favorable mRS 17%; radiographic vasospasm 45–55%); re-rupture further decreased the favorable mRS (14%) and led to universal EVD placement, despite small numbers. No IPR-RR events occurred in the HH III stratum, precluding comparison within this grade **(Table ****2**,S1**)**.

**Table 2:** Distribution of favorable discharge outcome (mRS ≤ 2) by intraprocedural re-rupture (IPR-RR) status within each Hunt–Hess (HH) grade. IPR-RR status

	HH I (*n* = 126)	HH II (*n* = 212)	HH III (*n* = 185)	HH IV (*n* = 111)	HH V (*n* = 58)
Absent (–)	85 / 125 68.0 %	140 / 206 68.0 %	98 / 185 53.0 %	23 / 104 22.1 %	6 / 56 10.7 %
Present (+)	1 / 1 100 %	2 / 6 33.3 %	—	2 / 7 28.6 %	0 / 2 0 %

Values are expressed as the “number of patients with the favorable outcome / total patients in that stratum," followed by the corresponding percentage

An em-dash (—) indicates that no IPR-RR events occurred in the HH III group, precluding percentage calculation

### Rescue management and outcomes

Among 17 IPR-RR events, coil packing was performed in 17/17 (100%), protamine was administered in 17/17 (100%), and balloon inflation across the neck was used in 14/17 (82%). Favorable discharge outcome (mRS ≤ 2) occurred in 5/17 (29.4%), while 12/17 (70.6%) had unfavorable outcomes. Mean age was 42.0 ± 17.5 years in the favorable group versus 52.8 ± 11.7 years in the unfavorable group. Favorable outcomes were observed in 3/13 (23.1%) females and 2/4 (50%) males. Hypertension was present in 3/9 (33.3%) favorable versus 6/9 (66.7%) unfavorable cases. Hyperlipidemia was present in 2/2 favorable and 0/2 unfavorable cases. ACoM location accounted for 4/5 (80%) favorable versus 2/12 (16.7%) unfavorable outcomes. Balloon inflation was performed in 5/5 (100%) favorable versus 9/12 (75%) unfavorable cases. Raymond–Roy class I occlusion was achieved in 5/5 (100%) favorable versus 3/12 (25%) unfavorable cases **(**Tables 3 and 4**)**.

**Table 3:** Rescue maneuvers performed after intraprocedural re-rupture (IPR-RR) of ruptured intracranial aneurysms

Rescue maneuver	*n* (%)^†^	Technical note
Coil packing / rapid continuation of offending coil	17 (100)	Index coil completed and additional soft coils deployed to seal the rent
Balloon inflation across the neck	14 (82)	Balloon was pre-positioned in every case; inflation achieved hemostasis.
Immediate protamine bolus (50 mg)	17 (100)	Reversed anticoagulation.

*Multiple maneuvers were often used in the same case; frequencies therefore sum to > 100%

†Percentages calculated using 17 IPR-RR events as the denominator

**Table 4:** Intraprocedural-rupture cohort (*N* = 17) – baseline factors vs. discharge outcome

Variable	Category	Favorable (%)	Unfavorable (%)
Age (yrs)	Mean	42.0 ± 17.5	52.8 ± 11.7
Sex	Female	3 (17.6)	10 (58.8)
	Male	2 (11.8)	2 (11.8)
Smoking	Never	0	4 (23.5)
	Prior	1 (5.9)	3 (17.6)
	Current	3 (17.6)	3 (17.6)
Hypertension	Yes	3 (17.6)	6 (35.3)
	No	2 (11.8)	4 (23.5)
Hyperlipidemia	Yes	2 (11.8)	0
	No	3 (17.6)	10 (58.8)
Family history	Yes	1 (5.9)	1 (5.9)
	No	4 (23.5)	9 (52.9)
Etiology	Idiopathic	5 (29.4)	11 (64.7)
	Dissecting	0	1 (5.9)
Location	ACoM	4 (23.5)	2 (11.8)
	MCA bifurcation	0	2 (11.8)
	Ophthalmic	0	1 (5.9)
	PCoM	1 (5.9)	4 (23.5)
	Others	0	3 (17.6)
Treatment technique	BAC	5 (29.4)	9 (52.9)
	Simple coiling	0	2 (11.8)
	Flow-diverter	0	1 (5.9)
Balloon inflated across the neck.	Yes	5 (29.4)	9 (52.9)
	No	0	3 (17.6)
Raymond–Roy class	I	5 (29.4)	3 (17.6)
	II	0	5 (29.4)
	III	0	4 (23.5)

## Discussion

In this cohort of 702 ruptured aneurysm treatments, IPR-RR occurred in 2.4% of cases and was more frequently observed in younger patients and in aneurysms larger than 10 mm. IPR-RR was associated with lower rates of favorable discharge functional outcome (29% vs. 52%) but similar in-hospital mortality (18% vs. 10%). Across Hunt–Hess strata, IPR-RR was accompanied by higher rates of vasospasm and greater use of external ventricular drainage. All rupture events were managed with immediate coil packing and protamine reversal, with balloon inflation used in 82% of cases.

Interestingly, the higher frequency of IPR-RR observed in larger aneurysms in our cohort contrasts with prior literature, which has consistently identified small aneurysm size as a major risk factor for intraprocedural rupture [7]. Several factors may explain this discrepancy. Procedural mechanics likely differ between small and large aneurysms: while very small aneurysms are more susceptible to perforation during initial microcatheter or wire manipulation [7], larger aneurysms typically require more extensive coil deployment, higher coil mass, and repeated catheter repositioning, which may increase wall stress and predispose to rupture during later stages of embolization [1, 2, 3]. In addition, treatment strategies vary with aneurysm size, as larger lesions more often require adjunctive techniques such as balloon remodeling or complex coil constructs, which may introduce additional mechanical forces or transient hemodynamic changes [7]. Selection and referral patterns may also contribute, as larger or more complex aneurysms treated at high-volume centers may not reflect those included in prior series. Collectively, these observations suggest that rupture risk may be influenced not only by aneurysm size itself but also by the interaction between aneurysm morphology and procedural complexity.

IPR complicates intracranial aneurysm treatment, differing in impact based on whether the aneurysm is ruptured or unruptured. IPR during coiling of unruptured aneurysms is usually less severe due to controlled conditions [3], whereas IPR in ruptured cases results in significantly worse outcomes [1]. Although surgical clipping has a higher rate of IPR, it allows for immediate hemostasis through temporary artery occlusion, bipolar coagulation, and dome clipping [11] and affords the advantage of having an open-to-air system wherein acute blood can be suctioned and removed actively to prevent devastating increases in intracranial pressure (ICP). Patient manipulation to improve venous return, such as raising the head above the heart, is also available in the OR. In contrast, endovascular management of IPR occurs with the patient’s head of bed flat and in an entirely contained system, resulting in rapidly raised ICP and relying on quick coil packing, balloon inflation, reversing anticoagulation, or, in severe cases, occluding the parent artery [12]; surgical intervention may be necessary if hemostasis fails [13]. Although balloon-assisted coiling was more frequently used in IPR-RR cases, it was not significantly associated with rupture in our cohort. This likely reflects its preferential use in more complex aneurysms rather than a direct causal relationship. Prior studies have suggested that balloon inflation may alter intra-aneurysmal hemodynamics or impose mechanical stress on a fragile dome; however, in contemporary practice, balloon techniques also serve a critical protective role by enabling rapid flow arrest and facilitating hemostasis during rupture events. Thus, balloon-assisted techniques should be interpreted as context-dependent, reflecting both procedural complexity and an essential tool for intraprocedural rescue rather than an independent risk factor [14].

Managing IPR during endovascular coiling of ruptured intracranial aneurysms presents unique challenges due to limited access and a constrained environment. Immediate steps include reversing heparin with protamine sulfate and continuing coil delivery to pack the rupture site until contrast extravasation ceases. Adjuncts such as proximal balloon inflation or manual carotid compression may provide temporary hemostasis [15]; if bleeding continues, emergency surgical decompression or parent artery occlusion may be necessary [13]. Balloon remodeling effectively manages aneurysm rupture during procedures. Santillan et al. [16] reported that a pre-positioned balloon achieved immediate hemostasis in 13 of 22 intraprocedural ruptures, reducing the rate of WFNS worsening from 55.5% to 7.7% upon inflation (*P* = 0.023). Based on this, a compliant balloon was positioned across the aneurysm neck in high-risk cases, and inflation was performed in 82% of 17 events to seal the defect within 30 s while coil packing continued. A timer was activated on the screen for documentation purposes, and inflation was limited to under 5 min, with brief deflations performed to confirm cessation of bleeding, minimize thromboembolic risk, and ensure contrast visibility for coil detachment.

Unlike surgical clipping, which permits immediate bleeding control and CSF diversion via open ventriculostomy, the endovascular approach often requires separate and delayed EVD placement, complicating acute intracranial hypertension management. Delays in controlling intracranial pressure after IPR-RR can worsen secondary brain injury from prolonged impaired cerebral perfusion and hydrocephalic tension, leading to poorer outcomes. Dalfino et al. noted that small, ruptured aneurysms, especially when manipulated with wires or stiff catheters, are at risk of rupture; microcatheter navigation can help prevent dome perforation in these delicate lesions [17]. Additionally, Stapleton et al. found that IPR cases have significantly higher EVD and permanent shunt placement rates than uncomplicated coiling [1], highlighting the challenges of managing acute hydrocephalus in the endovascular suite. This difference emphasizes the need for pre-procedural risk stratification to address IPR-related complications. In high-risk cases, staging an EVD kit in the angiography suite and having a neurosurgical team on standby significantly reduces time from IPR to CSF diversion, minimizing secondary hydrocephalic injury. Hybrid operating rooms provide an optimal safety net: their combined endovascular and microsurgical capabilities allow for swift conversion to open clipping or decompression—including rapid EVD placement—when IPR-RR exceeds percutaneous control. Integrating such versatile environments into aneurysm programs could significantly shorten rescue times and mitigate secondary neurological damage.

**Fig. 3:**
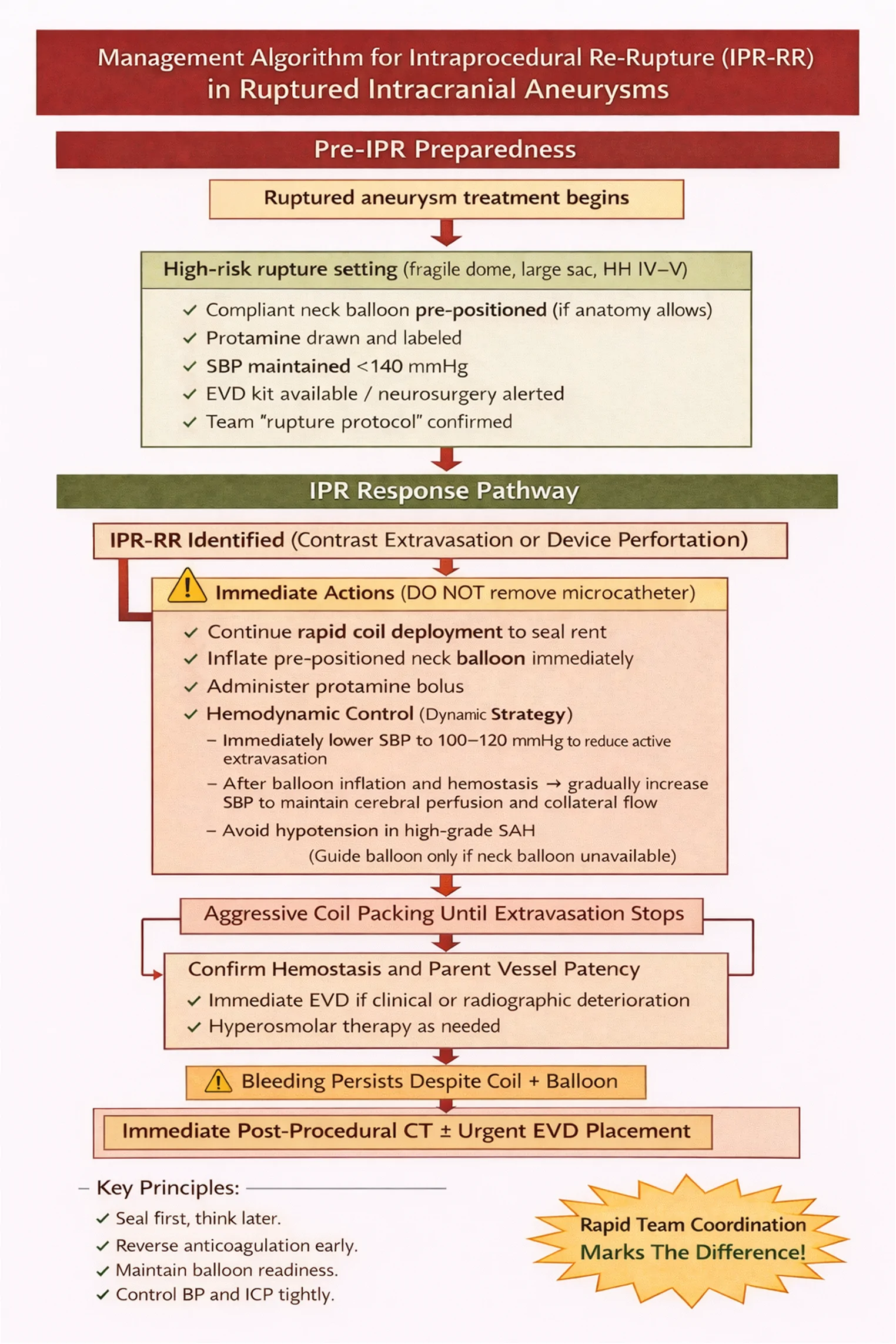
Algorithm for management of intraprocedural re-rupture in ruptured aneurysms, detailing preparedness, immediate hemostatic measures, and post-rupture critical care steps

Moreover, here are practical steps that may help optimize the management of IPR-RR. Before catheter work begins, the following preparedness measures are recommended: (i) ACT and platelet-function results should be verified; (ii) the subsequent coil should be kept ready during deployment and the planned framing coil should be opened and prepared prior to accessing the aneurysm; (iii) a compliant balloon should be parked across the neck when anatomy allows; (iv) the anesthesia team should be briefed to maintain systolic BP < 140 mmHg; (v) protamine 50 mg should be drawn and labeled; and (vi) a verbal cue should be agreed upon—at the word “rupture,” protamine is administered by the anesthesia team while coiling is continued by the operator. Adapted from standard cerebrovascular manuals, this protocol has markedly reduced procedural confusion and IPR-RR morbidity in our practice **(**Fig. 3**)**.

Our findings show that IPR-RR worsens the peri-procedural course across all HH strata, a result noted but not fully explored in prior literature. In our cohort, low-grade SAH patients (HH 1–2) without IPR-RR had a favorable discharge outcome in 66% of cases, which fell to 43% with IPR-RR. EVD requirement and vasospasm rates nearly doubled. High-grade patients (HH 4–5) fared worse: IPR-RR resulted in universal EVD use and only 14% favorable outcomes. Stapleton et al. similarly reported that IPR in ruptured aneurysms significantly increased the need for CSF diversion (EVD and shunting) and symptomatic vasospasm rates, with no statistical difference in long-term functional outcomes [1]. Park et al. supported this, noting longer ICU stays and worse discharge mRS in patients with IPR during ruptured aneurysm treatment [4]. Moreover, Zhang et al. observed a 33.3% mortality rate following IPR in ruptured aneurysms, highlighting the acute severity of this event [6].

This study has several limitations. First, its retrospective, single-center design limits generalizability and introduces the potential for selection and institutional practice bias. Second, the low number of IPR-RR events (*n* = 17), while reflecting the rarity of this complication, limits statistical power and precludes reliable multivariable modeling; therefore, our findings should be interpreted with caution and are intended for descriptive purposes only. Third, angiographic identification of IPR-RR may underestimate minor or self-limited rupture events that are not clearly visualized, potentially leading to underreporting.

In addition, procedural factors such as operator experience, device selection, and intra-procedural decision-making were not fully standardized or captured in a manner that allows detailed analysis, which may influence both the occurrence and management of IPR-RR. Outcome measures were limited to in-hospital endpoints, and discharge mRS may be confounded by baseline hemorrhage severity, medical complications, and variations in post-procedural care. Finally, given the observational design, causal relationships cannot be established, and unmeasured confounders may have influenced both the occurrence of IPR-RR and clinical outcomes.

Given the rarity of IPR-RR, multicenter and international registries will be essential to enable adequately powered predictive analyses.

## Conclusion

Intraprocedural re-rupture occurred in 2.4% of ruptured aneurysm treatments in this single-center cohort and was linked to significant early morbidity. It appeared across various aneurysm sizes and severities, often requiring immediate rescue such as coil packing, balloon inflation, and anticoagulation reversal. Due to the risk of rapid hemodynamic deterioration, maintaining readiness—such as pre-positioned balloons, rapid heparin reversal, strict blood pressure control, and cerebrospinal fluid diversion—is essential to reduce secondary neurological injury. Careful microcatheter handling, controlled coil deployment, and coordinated team effort may help lower the impact of this rare but serious complication.

## Supplementary information

Below is the link to the electronic supplementary material.

Supplementary File 1 (DOCX 17.8 KB)

## Data Availability

All data generated or analyzed during this study are available upon the request from the editors.
