# Supplementary material for: Intraprocedural re-rupture during endovascular treatment of ruptured intracranial aneurysms: A single-center experience
Source: Neuroradiology. 2026 May 20;68(8):2305–13. doi: 10.1007/s00234-026-04022-8 (PMC13578067; doi:10.1007/s00234-026-04022-8)
Supplement: Supplementary file 1 [file 234_2026_4022_MOESM1_ESM.docx]

**Table S1**. Peri-procedural course broken down by admission Hunt–Hess grade (HH) and IPR-RR status

| HH | IPR | Favorable outcome | Unfavorable outcome | EVD in placed | | EVD timing† | | | Radiographic vasospasm‡ | | | Symptomatic vasospasm | |
| --- | --- | --- | --- | --- | --- | --- | --- | --- | --- | --- | --- | --- | --- |
| Grades | **Yes, vs No** | **mRS ≤ 2** **n (%)** | **mRS > 2** **n (%)** | **Yes** | **No** | **Prior** | **After** | **None** | **Yes** | **No** | **NE** | **Yes** | **No** |
| 1 | No | 85 (68.0) | 40 (32.0) | 37 (29.6) | 88 (70.4) | 31 (24.8) | 5 (4.0) | 1 (0.8) | 30 (24.0) | 91 (72.8) | 4 (3.2) | 17 (13.6) | 108 (86.4) |
|  | Yes | 1 (100) | — | 1 (100) | — | — | — | 1 (100) | 1 (100) | — | — | — | 1 (100) |
| 2 | No | 140 (68.0) | 66 (32.0) | 76 (36.9) | 130 (63.1) | 67 (32.5) | 7 (3.4) | 3 (1.5) | 56 (27.2) | 143 (69.4) | 6 (2.9) | 30 (14.6) | 170 (82.5) |
|  | Yes | 2 (33.3) | 4 (66.7) | 2 (33.3) | 4 (66.7) | 2 (33.3) | — | — | 3 (50.0) | 3 (50.0) | — | 2 (33.3) | 4 (66.7) |
| 3 | No | 98 (53.0) | 87 (47.0) | 145 (78.4) | 39 (21.1) | 131 (70.8) | 14 (7.6) | — | 71 (38.4) | 111 (60.0) | 3 (1.6) | 46 (24.9) | 139 (75.1) |
|  | Yes | — | — | — | — | — | — | — | — | — | — | — | — |
| 4 | No | 23 (22.1) | 81 (77.9) | 93 (89.4) | 11 (10.6) | 86 (82.7) | 7 (6.7) | — | 47 (45.2) | 56 (53.8) | — | 38 (36.5) | 66 (63.5) |
|  | Yes | 2 (28.6) | 5 (71.4) | 7 (100) | — | 6 (85.7) | 1 (14.3) | — | 5 (71.4) | 2 (28.6) | — | 1 (14.3) | 6 (85.7) |
| 5 | No | 6 (10.7) | 50 (89.3) | 54 (96.4) | 2 (3.6) | 52 (92.9) | 2 (3.6) | — | 23 (41.1) | 31 (55.4) | 1 (1.8) | 15 (26.8) | 39 (69.6) |
|  | Yes | — | 2 (100) | 2 (100) | — | 2 (100) | — | — | 1 (50.0) | 1 (50.0) | — | 1 (50.0) | 1 (50.0) |
